# Interleukin-8: A potent promoter of human lymphatic endothelial cell growth in gastric cancer

**DOI:** 10.3892/or.2015.3916

**Published:** 2015-04-17

**Authors:** JUN SHI, YONG-JIN LI, BING YAN, PIN-KANG WEI

**Affiliations:** Department of Traditional Chinese Medicine, Shanghai Changzheng Hospital, The Second Military Medical University, Shanghai, P.R. China

**Keywords:** interleukin-8, lymphangiogenesis, gastric cancer, lymphatic vessel endothelial hyaluronic acid receptor 1, lymph-angiogenic growth factor

## Abstract

Lymphatic metastasis is a major progression route of gastric cancer. Interleukin-8 (IL-8), as an inflammatory cytokine, is induced by *Helicobacter pylori* infection and is strongly associated with gastric cancer development and metastasis. The blood and lymphatic systems are similar in their function and gene expression profiles. It has been proposed that IL-8 activates angiogenesis. However, the direct role of IL-8 in lymphangiogenesis in gastric cancer remains unclear. We investigated the effect of IL-8 on the growth of human lymphatic endothelial cells (LECs). In addition, protein and mRNA expression of selected lymphangiogenesis markers was assessed in these cells. LECs were co-cultured with gastric cancer SGC7901 cells and exposed to various concentrations of IL-8 (0, 0.2, 0.5, 0.8 and 1.0 ng/ml). The Cell Counting Kit-8 was used to evaluate LEC proliferation (cultured for 1-6 days). Then, protein (immunofluorescence and western blotting) and mRNA [quantitative transcription-polymerase chain reaction (qPCR)] levels were measured in samples obtained from the 24-h cultured cells, for lymphatic vessel endothelial hyaluronic acid receptor-1 (LYVE-1), vascular endothelial growth factor (VEGF)-C, VEGF-D and vascular endothelial growth factor receptor-3 (VEGFR-3). The data presented herein demonstrated that IL-8 promotes the proliferation of LECs and enhances the protein and mRNA expression of LYVE-1. Notably, IL-8 inhibited VEGF-C, VEGF-D and VEGFR-3 protein expression as well as VEGF-D and VEGFR-3 mRNA expression. These findings suggest that IL-8 may be a potent inducer of LECs, although this effect does not appear to involve the VEGF-C/VEGF-D and VEGFR-3 signaling pathway.

## Introduction

Gastric cancer is the leading cause of death from gastrointestinal malignancy and is associated with *Helicobacter pylori* (Hp) infection. Higher levels of interleukin-8 (IL-8), a CXC chemokine, have been shown in Hp-infected gastric tissues in comparison with Hp-negative tissues (1). Meanwhile, upregulation of IL-8 was observed in gastric cancer (2), and IL-8 plays an important role in adhesion, migration and invasion of gastric cancer cells (3). Hence, overexpression of IL-8 is associated with the development and metastasis of gastric cancer (4).

Gastric cancer patients (50–75%) are diagnosed at stages III or IV with lymphatic metastasis (5), which is considered the strongest prognostic factor regarding long-term survival in gastric cancer. Lymphangiogenesis, the formation of lymphatic vessels, is linked to lymphatic metastasis and plays an important role in malignant cell dissemination. The growth of lymphatic endothelial cells (LECs) is regarded as the fundamental step of lymphangiogenesis. The blood and lymphatic systems as the two major circulatory systems in humans are similar in their function and anatomy. Moreover, the endothelial cells from blood and lymphatic vessels display similar gene expression profiles; many growth factors and various inflammatory mediators were shown to activate both angiogenesis and lymphangiogenesis (6–8). Notably, the inflammatory cytokine IL-8 has been shown to activate angiogenesis (9,10). However, the direct role of IL-8 in gastric cancer lymphangiogenesis is poorly understood. Furthermore, the recent identification of lymphatic endothelial-specific markers has greatly increased attention to the regulation of lymphangiogenesis in the cancer microenvironment. It has been reported that vascular endothelial growth factor (VEGF)-C and VEGF-D both bind their receptor, vascular endothelial growth factor receptor-3 (VEGFR-3), and VEGF signaling is involved in the development and growth of the lymphatic system (11). It has been shown that secretion of VEGF-C and VEGF-D by some tumors induces VEGFR-3 activation in the vascular endothelium, thereby promoting the formation of new lymphatic vessels (12). Whether VEGF signaling is involved in IL-8-induced lymphangiogenesis in gastric cancer is still unclear. In the present study, we evaluated the effect of IL-8 on the growth of LECs and expression of VEGF-C, VEGF-D and VEGFR-3, using a co-culture model including gastric cancer SGC7901 cells and LECs.

## Materials and methods

### Cell culture

The human gastric cancer SGC7901 and human lymphatic endothelial cells were purchased from the Cell Bank of the Chinese Academy of Sciences (Shanghai, China) and ScienCell Research Laboratories (Carlsbad, CA, USA), respectively. All cells were cultured in endothelial cell medium (ScienCell), supplemented with 5% fetal bovine serum (FBS; Zhejiang Tianhang Biological Technology Co., Ltd., Hangzhou, China), 1% penicillin/streptomycin and 1% endothelial cell growth supplement (ScienCell). Cells were maintained at 37°C in a humidified chamber containing 5% CO_2_.

### Co-culture model, cell grouping and IL-8 treatment

SGC7901 cells (2×10^5^ cells/well) were cultured for 24 h with an IL-8 stock solution (Sigma-Aldrich, St. Louis, MO, USA) added to a predetermined concentration. Then, SGC7901 cell culture media were collected and added to LECs for further incubation. Based on culture media and IL-8 doses, 6 groups were established experimentally: control group (only endothelial cell medium without SGC7901 cell culture medium) and 5 IL-8 groups with SGC7901 cell culture media containing various concentrations of the cytokine (0, 0.2, 0.5, 0.8 and 1 ng/ml groups).

### Cell proliferation assay

Cell proliferation was assessed using a Cell Counting Kit-8 (CCK-8; Dojindo, Kunamoto, Japan), according to the manufacturer's instructions. Briefly, LECs (2×10^3^ cells/well) in logarithmic phase were cultured in 96-well plates and incubated overnight to allow adherence. After washing, various culture media (see cell grouping above) were added to the LECs, followed by 1–6 days of incubation. There were 9 replicate wells for each group. At each time point, 10 *μ*l WST-8, which produces a water-soluble formazan, was diluted in 100 *μ*l endothelial cell medium and added to the LECs. After an additional 3 h incubation of the LECs, absorbance was measured by a microplate reader (Multiskan MK3; Thermo Fisher, Waltham, MA, USA) at 450 nm to obtain an optical density (OD) value. OD ultimate value = OD measured value - OD blank value.

### Immunofluorescence staining

LECs (5×10^4^) were seeded on coverslips in 24-well plates and cultured with endothelial cell medium at 37°C to allow adherence. Then LECs were cultured in the presence of various culture media containing different IL-8 concentrations for 24 h. After fixation in 4% paraformaldehyde for 15 min, sequential treatments with 0.5% Triton X-100 for 10 min and 4% bovine serum albumin (BSA) for 1 h at room temperature, LECs were incubated with lymphatic vessel endothelial hyaluronic acid receptor-1 (LYVE-1) rabbit polyclonal antibody (1:50), VEGF-C goat polyclonal antibody (1:80) (both from Santa Cruz Biotechnology, Santa Cruz, CA, USA), VEGF-D rabbit monoclonal antibody (1:100; Epitomics, Burlingame, CA, USA), VEGFR-3 rabbit polyclonal antibody (1:100; Abcam, Cambridge, UK), respectively, at 4°C overnight. Afterwards, Cy3-conjugated Affinipure goat anti-rabbit IgG (H+L) (1:1,000 dilution) and Cy3-conjugated Affinipure donkey anti-goat IgG (H+L) (1:1,000 dilution) (both from Proteintech Group, Wuhan, China) were added to the corresponding samples for an additional 1 h. After counterstaining with DAPI, coverslips were observed under a laser confocal scanning microscope (LSM710; Zeiss, Oberkochen, Germany).

### Western blot analysis

LECs (2×10^5^) were incubated for 24 h, collected and lysed in 150 *μ*l cell lysate buffer. Cell lysates were centrifuged for 1 min at 12,000 rpm, and the supernatants were collected, boiled in sample buffer for 10 min at 100°C and separated by SDS-PAGE (10% separation gel, 5% spacer gel). After electrotransfer onto polyvinylidene difluoride film (Bio-Rad, Hercules, CA, USA), the membranes were blocked for 1 h at room temperature. Then, LYVE-1 rabbit polyclonal antibody (1:250), VEGF-C goat polyclonal antibody (1:250), VEGF-D rabbit monoclonal antibody (1:250), VEGFR-3 rabbit polyclonal antibody (1:250) and GAPDH mouse monoclonal antibody (1:3,000; Sungene, Tianjin, China) were used to probe the blots overnight at 4°C. After a washing step, the membranes were incubated with anti-rabbit IgG-HRP (1:1,000), anti-mouse IgG-HRP (1:3,000) and anti-goat IgG-HRP (1:1,000) secondary antibodies, respectively, for 1 h at room temperature. The membranes were washed and detection was carried out using a SuperSignal West Pico Chemiluminescent Substrate kit (Thermo Fisher Scientific). Finally, the membranes were exposed to X-ray film in a darkroom and scanned by an image analyzer. Grayscale analysis was performed by ImageJ software, and LYVE-1, VEGF-C, VEGF-D and VEGFR-3 levels were assessed, relative to GAPDH (internal control). The rates of the 5 experimental groups were normalized to the control group (values set at 1) and considered protein levels. All experiments were repeated three times.

### Quantitative transcription-polymerase chain reaction (qPCR) analysis

LECs were seeded in 12-well plates at a density of 7×10^4^ cells/well and incubated for 24 h. Total RNA was extracted from the LECs using TRIzol reagent (Takara, Shiga, Japan) and reverse transcribed with the RevertAid Reverse Transcriptase (Thermo Fisher Scientific), according to the manufacturer's instructions. Quantitative RT-PCR was performed with SYBR-Green chemistry on a Bio-Rad iQ5 Real-Time PCR system (Bio-Rad), with each sample analyzed in triplicate. The reaction conditions consisted of one cycle of 95°C for 2 min, 95°C for 15 sec, 60°C for 20 sec, 72°C for 20 sec and then 40 cycles of 72°C for 30 sec. Relative levels of LYVE-1, VEGF-C, VEGF-D and VEGFR-3 mRNA expression were normalized to GAPDH mRNA expression, and calculated by the 2^−ΔΔCt^ method. The primer sequences for each gene analyzed are summarized in Table I.

### Statistical methods

The resulting measurements were expressed as mean ± standard deviation (SD) for each data point. All data were analyzed using the SPSS 13.0 software. ANOVA for repeated measurement data was used to determine statistical significance of differences in the cell proliferation experiments. One-way ANOVA was used to assess the expression of protein or mRNA. The LSD method was used to analyze *post-hoc* multiple comparisons. All reported P-values were two-sided with a statistical significance level of <0.05.

## Results

### IL-8 promotes the proliferation of human LECs

**Figure 1:**
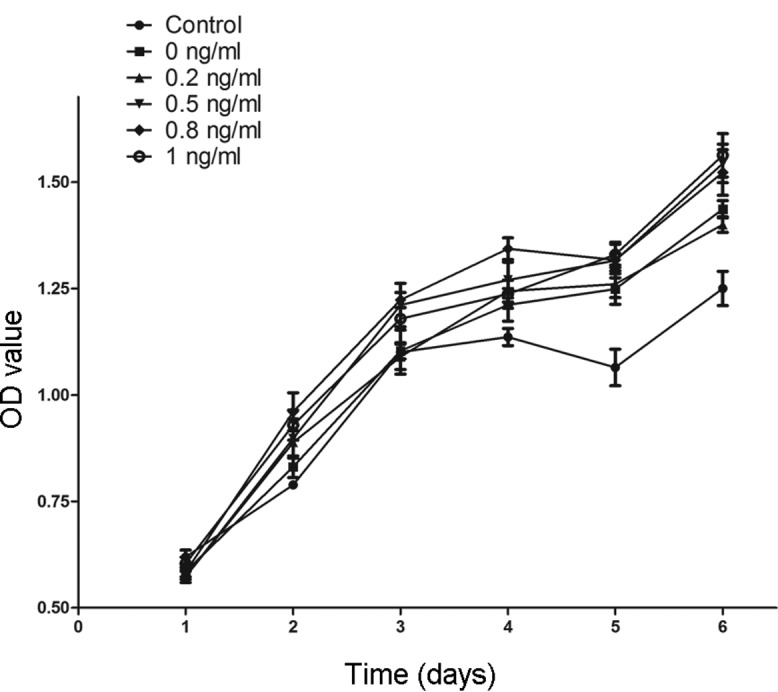
IL-8 promotes proliferation of LECs. From the first to the sixth day, OD values increased gradually. Cell proliferation was significantly different at various time points (P<0.05), and an interaction was found between time factor and intervention (P<0.05). Growth of the 0 ng/ml group cultured with pure SGC7901 cell culture medium was faster than that of the control group (P<0.05). Meanwhile, OD values obtained for the 0.5, 0.8 and 1.0 ng/ml groups were significantly higher when compared with the OD value of the 0 ng/ml group (P≤0.001). IL-8, interleukin-8; LECs, lymphatic endothelial cells; OD, optical density.

We investigated the effect of IL-8 on LEC proliferation using the CCK-8 kit. Our data showed a significant difference in the proliferation of cells between interventions and days. Pure SGC7901 cell culture medium promoted proliferation of the LECs. Notably, IL-8 at concentrations >0.5 ng/ml potently induced LEC growth (Table II, Fig. 1).

### IL-8 promotes LYVE-1 protein and mRNA expression

**Figure 2:**
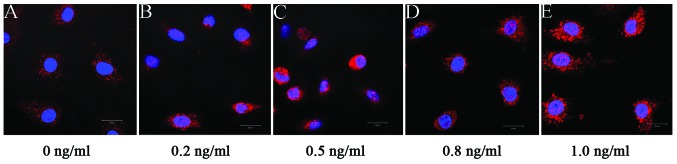
IL-8 promotes LYVE-1 protein expression in LECs (immunofluorescence staining, ×630). Cell nuclei counterstained with DAPI (blue) and LYVE-1 stained with Cy3 (red). LYVE-1 protein was found on the cell membrane and in the cytoplasm. IL-8, interleukin-8; LYVE-1, lymphatic vessel endothelial hyaluronic acid receptor-1; LECs, lymphatic endothelial cells. (A) 0, (B) 0.2, (C) 0.5, (D) 0.8 and (E) 1.0 ng/ml group.

**Figure 3:**
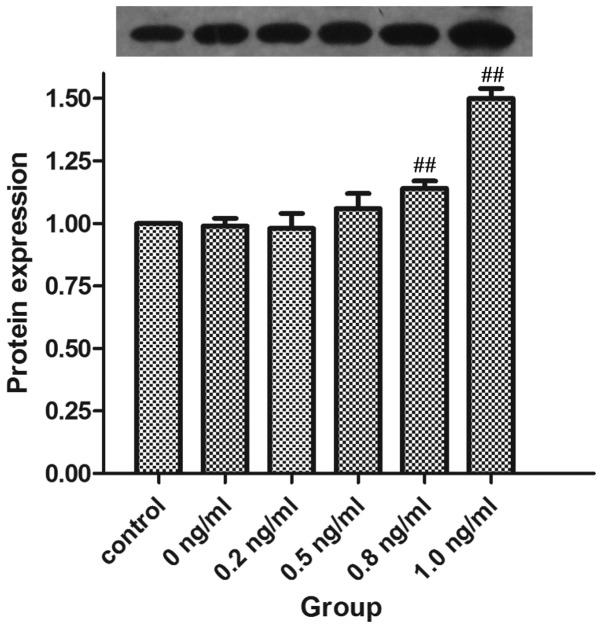
IL-8 promotes LYVE-1 protein expression in LECs (western blotting, 40 kDa). The expression levels in the 0 ng/ml group were not significantly different when compared with the values obtained for the control group (P=1.000). In contrast, the expression levels in 0.8 and 1.0 ng/ml groups were significantly different to that of the 0 ng/ml group (P=0.009 and P<0.001, respectively). ^##^P<0.01 vs. 0 ng/ml group. IL-8, interleukin-8; LYVE-1, lymphatic vessel endothelial hyaluronic acid receptor-1; LECs, lymphatic endothelial cells.

We next investigated the effect of IL-8 on protein and mRNA expression of LYVE-1, a marker of lymphogenesis. Pure SGC7901 cell culture medium did not alter LYVE-1 protein expression levels. However, IL-8 promoted LYVE-1 protein expression at concentrations >0.8 ng/ml (Table III, Figs. 2 and 3).

**Figure 4:**
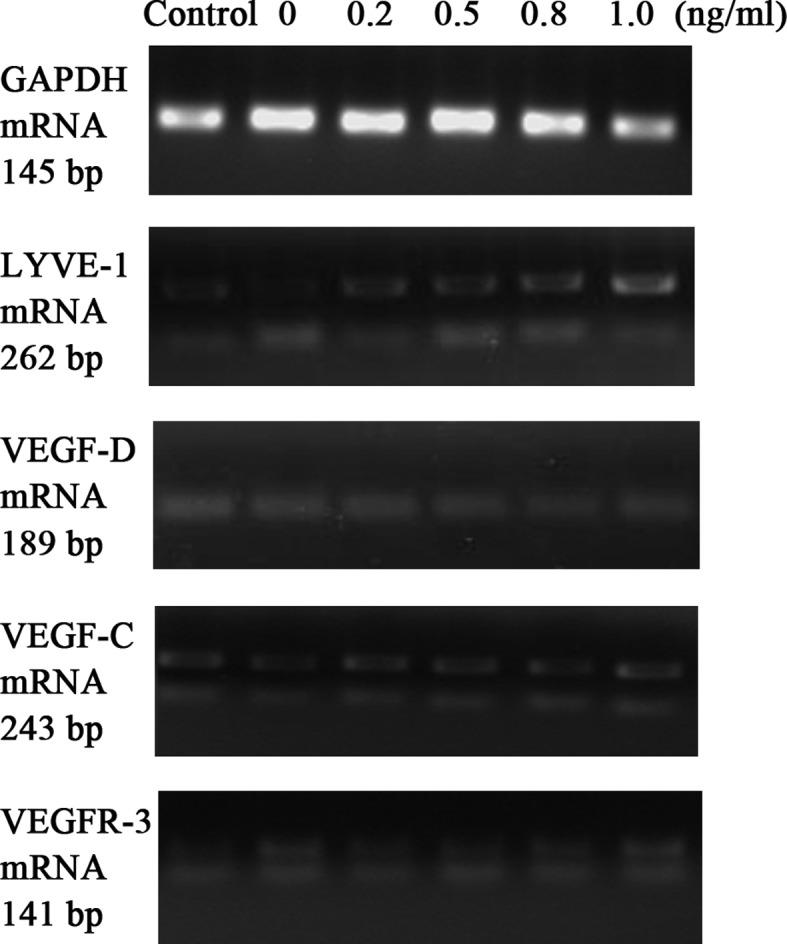
IL-8 inhibits VEGF-D and VEGFR-3 mRNA expression in LECs (qPCR). A non-significant difference in VEGF-C mRNA levels was found among all groups (P=0.079). Pure SGC7901 cell culture medium inhibited VEGF-D mRNA expression (P<0.001) and promoted VEGFR-3 mRNA expression (P<0.001). VEGF-D mRNA expression levels in the 0.5, 0.8 and 1.0 ng/ml groups were significantly decreased compared with the 0 ng/ml group (P=0.024, P<0.001 and P=0.004, respectively). A similar decreased expression of VEGFR-3 mRNA in the 0.2, 0.5, 0.8 and 1.0 ng/ml groups was observed (P<0.001, P=0.001, P=0.001 and P=0.001, respectively). IL-8, interleukin-8; VEGF-D, vascular endothelial growth factor-D; VEGFR-3, vascular endothelial growth factor receptor-3; LECs, lymphatic endothelial cells; VEGF-C, vascular endothelial growth factor-C.

Similar results were found for mRNA expression of LYVE-1; LYVE-1 mRNA levels were not regulated by pure SGC7901 cell culture medium and were upregulated by IL-8 concentrations >0.5 ng/ml (Table IV, Fig. 4).

### IL-8 inhibits protein and mRNA expression of VEGF-D, VEGF-C and VEGFR-3

**Figure 5:**
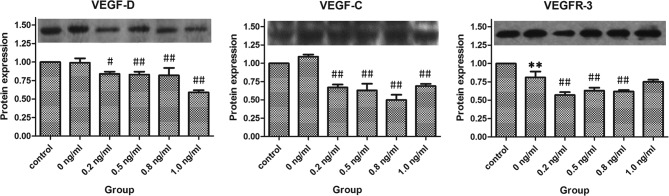
IL-8 inhibits VEGF-D, VEGF-C and VEGFR-3 protein expression in LECs (western blotting, VEGF-D 40 kDa, VEGF-C 80 kDa, VEGFR-3 146 kDa). The VEGF-D and VEGF-C protein expression levels in the 0 ng/ml group showed a non-statistically significant difference compared with the control group (P=0.817 and P=0.259, respectively). However, the VEGFR-3 protein expression was inhibited by pure SGC7901 cell culture medium (P<0.001). The expression levels of VEGF-D in the 0.2, 0.5, 0.8 and 1.0 ng/ml groups were all significantly decreased compared with the 0 ng/ml group (P=0.030, P=0.002, P=0.001 and P<0.001, respectively). A similar downregulated expression of the VEGF-C protein was observed (all P<0.001). Meanwhile, VEGFR-3 expression levels in the 0.2, 0.5 and 0.8 ng/ml groups were significantly decreased compared with the 0 ng/ml group (P<0.001, P=0.002 and P=0.001, respectively). **P<0.01 vs. control group; ^#^P<0.05, ^##^P<0.01 vs. 0 ng/ml group. L-8, interleukin-8; VEGF-D, vascular endothelial growth factor-D; VEGF-C, vascular endothelial growth factor-C; VEGFR-3, vascular endothelial growth factor receptor-3; LECs, lymphatic endothelial cells.

The secretion of VEGF-C and VEGF-D was reported to induce the formation of new lymphatic vessels via activation of VEGFR-3 (13). Hence, we next investigated the possible role of VEGF-C/VEGF-D and the VEGFR-3 signaling pathway in IL-8-induced LEC growth. We found that pure SGC7901 cell culture medium had an insignificant effect on VEGF-C and VEGF-D protein expression. This medium even inhibited VEGFR-3 protein expression. Notably, VEGF-C, VEGF-D and VEGFR-3 protein levels were overtly decreased after IL-8 addition (Table III, Fig. 5). Furthermore, we found that pure SGC7901 cell culture medium inhibited the expression of VEGF-D mRNA, yet enhanced VEGFR-3 gene expression. After IL-8 treatment, VEGF-D and VEGFR-3 mRNA expression levels were decreased, in good accordance with the protein levels described above. Neither pure SGC7901 cell culture medium nor IL-8 showed a significant effect on VEGF-C mRNA expression (Table IV, Fig. 4).

## Discussion

IL-8, a member of the neutrophil-specific CXC subfamily of chemokines, has been shown to be involved in leukocyte chemotaxis, inflammatory responses and infectious diseases (14). It plays an important role in the proliferation, invasion and migration of endothelial cells (15,16). Gastric cancer, the leading cause of death from gastrointestinal malignancy worldwide, is associated with *Helicobacter pylori* (Hp) infection (17,18). Hp directly increases gastric epithelial IL-8 protein secretion and IL-8 mRNA expression (1,19,20). Meanwhile, IL-8 upregulation also occurs in gastric cancer (2). Therefore, it has been suggested that IL-8, as a significant regulatory autocrine factor within the tumor microenvironment (21), is strongly associated with gastric cancer. In a previous study, we reported that recombinant IL-8 promotes adhesion, migration and invasion of human gastric cancer SGC7901 cells (22). Using cDNA and siRNA transfectants, Kuai *et al* reported similar findings (3). Furthermore, a previous study showed that IL-8 overexpression is linked to invasion and metastasis in gastric cancer (4). Therefore, IL-8 is considered as an important multifunctional factor in the metastasis of gastric cancer. The lymphatic system is a one way, open-ended complex network comprised of capillaries, trunks, ducts and lymph nodes. It is credited with the transport of tissue fluids, extravasated plasma proteins and cells back into the blood circulation. The lymphatic system is regarded as a fundamental route for cancer cell dissemination due to its initial lymphatic vessels larger than the blood capillaries, incomplete basement membrane and slower lymph flow than blood (23). Previous studies have found that lymphatic vessel density is correlated with the extent of lymph node metastasis (24–26). Meanwhile, expression of lymphangiogenic growth factors leads to formation of lymphatic vessels (24) and lymphatic metastasis can be blocked by inhibition of tumor lymphangiogenesis (27–29). Therefore, lymphangiogenesis is thought to play a pivotal role in cancer cells to format new lymphatic vessels and metastasis to the regional lymph nodes (30). In gastric cancer, lymphatic spread of tumor cells to regional lymph nodes is a common occurrence in the early stage (24,27). According to the American Joint Committee on Cancer (AJCC), lymph node metastasis of gastric cancer is one of the main prognostic factors; lymph node ratio has been proposed as a desirable predictor of survival (31). It has been revealed that lymphatic vessels refer to the spread of gastric cancer cells (24,27), and human gastric adenocarcinoma organizes neighboring lymphatic vessels by inducing tumor lymphangiogenesis (32), which is thought to contribute actively to the development of lymphatic metastasis in gastric cancer (27–29).

The blood and lymphatic systems are similar in their function and anatomy; endothelial cells from blood and lymphatic systems were reported to have similar gene expression profiles (6-8). In a number of studies, various pro-inflammatory cytokines were found to indirectly induce both angiogenesis and lymphangiogenesis by activation of infiltrating immune cells to secrete many lymph or angiogenic factors (33). Although IL-8, a pro-inflammatory cytokine with a function in cancer development and metastasis (4), was one of the first CXC-cytokines identified to activate angiogenesis (9,10), few studies have investigated its role in lymphangiogenesis. IL-8 was found to reduce post-surgical lymphedema formation by promoting lymphatic vessel regeneration via activation of lymphangiogenesis *in vitro* and *in vivo*. Notably, IL-8 directly promotes proliferation, tube formation and migration of cultured primary human LECs through its receptor CXCR2 (33). Mu *et al* revealed that IL-8 plays an important role in lysophosphatidic acid-induced lymphangiogenesis *in vitro* (34). In a study of prostate cancer xenografts, Sakai *et al* found that increased Bcl-2 expression enhanced the expression and secretion of key lymphangiogenic factors and increased serum IL-8 contents, which correlated with increased angiogenic and lymphangiogenic levels *in vivo* (35). However, the direct role of IL-8 in lymphangiogenesis in gastric cancer has not been established. In the present study, we hypothesized that IL-8 plays a critical role in gastric cancer lymphangiogenesis. We investigated the growth of LECs cultured in gastric cancer SGC7901 cell culture medium supplemented with IL-8. We found that IL-8 promotes the growth of LECs, suggesting that IL-8 may be a direct promoter of lymphangiogenesis in gastric cancer.

The identification of lymphatic endothelial-specific biological markers has greatly increased attention to the regulation of lymphangiogenesis in the cancer microenvironment. LYVE-1, a homolog of the hyaluronan receptor CD44, is a type I integral membrane polypeptide expressed on the cell surface as a 60-kDa protein. LYVE-1 is involved in hyaluronan metabolism in the lymphatic system where it transports hyaluronan from the extracellular matrix to lymph nodes (36,37). To date, LYVE-1 is one of the most widely used marker for LECs in normal and cancer tissues. Indeed, LCEs are routinely identified by the expression of LYVE-1. Based on the enhanced growth of LECs, we investigated the effect of IL-8 on LYVE-1 protein and mRNA expression. Consistent with the proliferation of LECs, IL-8 promoted LYVE-1 protein and mRNA expression, confirming IL-8 as a direct promoter of LECs.

The identification of vascular endothelial growth factors started with the discovery of VEGF-A in 1989 (38). To date, the VEGF family has been expanded to include VEGF-A, -B, -C, -D and -E and placental growth factor. VEGF-C not only stimulates mitosis and migration of endothelial cells, yet also increases vascular permeability. VEGF-D, structurally 48% identical to VEGF-C, is mitogenic to endothelial cells. Overexpression of VEGF-C/VEGF-D has been observed in a number of human types of cancers (25) and is involved in lymphatic hyperplasia (36,37), lymphatic vessel invasion and/or lymph node metastasis (24). Macrophages in the peritumoral stroma produce VEGF-C and VEGF-D in certain cancers to induce lymphangiogenesis (39). Moreover, previous studies have shown that various inflammatory factors, such as IL-7, induce VEGF-D expression and promote lymphangiogenesis via the c-Fos/c-Jun pathway in lung cancer (40). Three VEGF tyrosine kinase receptors have been identified to date: VEGFR-1, VEGFR-2 and VEGFR-3; both VEGF-C and VEGF-D bind VEGFR-3, a tyrosine kinase receptor mainly involved in lymphangiogenesis where it controls the development and growth of the lymphatic system; thus, the VEGF-C/VEGF-D and VEGFR-3 signaling pathway has been shown to play a central role in lymphangiogenesis, and is widely used in studies of lymphangiogenic signaling (24,25,27,41,42).

For gastric cancer, previous studies have indicated that the VEGF-C-VEGFR-3 signaling pathway plays a major role in lymphangiogenesis through both autocrine and paracrine mechanisms (43,44), and quantitative analysis of VEGF-C and VEGFR-3 may be useful in predicting metastasis to regional lymph nodes (30,45,46). The inhibition of VEGFR-3 phosphorylation is a therapeutic strategy for inhibiting lymph node metastasis of diffuse-type gastric cancer (47). However, the controversy remains as to whether VEGF-D is linked to lymphangiogenesis in gastric cancer. It was reported that both VEGF-C and VEGF-D enhanced lymphangiogenesis and neo-formation of lymphatic vessels in experimental gastric tumors via induction of VEGFR-3 expression (48). On the other hand, only the expression of VEGF-C (but not of VEGF-D) was significantly greater in gastric cancer patients with lymph node metastasis than in those without metastasis (49). We then investigated whether IL-8 promotes the growth of LECs by inducing the VEGF-C/VEGF-D and VEGFR-3 pathway. Notably. although enhanced growth of LECs and upregulated LYVE-1 expression were detected, VEGF-C, VEGF-D and VEGFR-3 were not upregulated and instead were inhibited by IL-8. Krzystek-Korpacka *et al* reported that elevation of circulating IL-8 was related to lymph node and distant metastases in esophageal squamous cell carcinomas, and circulating IL-8 correlated with lymphangiogenic VEGF-C rather than angiogenic VEGF-A. These findings suggested that IL-8 involvement in lymphatic spread may be indirect, with IL-8 acting by stimulating VEGF-C expression and secretion (50). Notable, the present study did not support such a conclusion in gastric cancer. We showed a direct effect of IL-8 on the growth of LECs without stimulating VEGF-C expression. In a previous study of post-surgical lymphedema, Choi *et al* investigated the possible role of VEGF-C-VEGFR-3 signaling in IL-8-induced LEC proliferation using chemical inhibitors (Ki8751 and MAZ51) for VEGF receptors. They found that VEGF signaling did not play a role in IL-8-activated LEC proliferation (33). Our results are in agreement with these findings. Enhanced growth of LECs accompanied decreased VEGF-C, VEGF-D and VEGFR-3 expression after IL-8 addition. Although inflammatory factors (e.g. IL-7) were reported to induce VEGF-D upregulation and promote lymphangiogenesis (40), we could not demonstrate the involvement of VEGF-D-VEGFR-3 signaling in IL-8-induced LEC growth. The interaction between IL-8 and VEGF-C/VEGF-D-VEGFR-3 signaling pathway remains therefore unclear. We previously found that IL-8 induced downregulated expression of VEGF-C, VEGF-D and VEGFR-3. IL-8 is a multifunctional inflammatory cytokine released through the nuclear factor (NF)-κB signaling pathway (51). Meanwhile, enhanced production of VEGF-C and VEGF-D was reported to be linked to NF-κB activation in inflammatory lymphangiogenesis (52). Thus, we suppose that the IL-8-induced inhibition of VEGF-C, VEGF-D and VEGFR-3 may be associated with the NF-κB signaling pathway. The mechanism of IL-8-induced lymphangiogenesis requires further investigation.

In conclusion, our data suggest IL-8 as a potent direct promoter of LEC growth in gastric cancer. However, this effect is not associated with VEGF-C/VEGF-D and VEGFR-3 signaling.

## Figures and Tables

**Table I:** Sequences of the primers used for qPCR.

mRNA	Sense primer sequence	bp
hGAPDH-F	5′-GGGTGTGAACCATGAGAAGTATG-3′	145
hGAPDH-R	5′-GATGGCATGGACTGTGGTCAT-3′	
LYVE-1-F	5′-ATCCCCTTACTCTACAATACCTGC-3′	262
LYVE-1-R	5′-GACATAGCAAAATCCAAGACCAG-3′	
VEGF-C-F	5′-GCTGGAGATGACTCAACAGATGG-3′	243
VEGF-C-R	5′-GGGGCAGGTTCTTTTACATACAC-3′	
VEGF-D-F	5′-CATCCCATCGGTCCACTAGG-3′	189
VEGF-D-R	5′-AGCCACCACATCGGAACAC-3′	
VEGFR-3-F	5′-TCATCGCTGTCTTCTTCTGGG-3′	141
VEGFR-3-R	5′-GGTATTCGCATTGCTCCTCC-3′	

LYVE-1, lymphatic vessel endothelial hyaluronic acid receptor-1; VEGF-C, vascular endothelial growth factor-C; VEGF-D, vascular endothelial growth factor-D; VEGFR-3, vascular endothelial growth factor receptor-3.

**Table II:** Effect of IL-8 on LEC proliferation (OD).

Group	1st Day	2nd Day	3rd Day	4th Day	5th Day	6th Day
Control	0.62±0.02	0.79±0.01	1.10±0.05	1.14±0.02	1.06±0.04	1.25±0.04
0 ng/ml	0.59±0.02	0.83±0.03	1.10±0.02	1.21±0.04	1.25±0.04	1.44±0.02
0.2 ng/ml	0.57±0.01	0.89±0.01	1.09±0.03	1.24±0.03	1.26±0.03	1.40±0.02
0.5 ng/ml	0.57±0.01	0.90±0.05	1.21±0.05	1.27±0.04	1.32±0.02	1.54±0.05
0.8 ng/ml	0.58±0.01	0.96±0.04	1.22±0.02	1.34±0.03	1.32±0.04	1.52±0.05
1.0 ng/ml	0.60±0.01	0.93±0.04	1.18±0.03	1.24±0.03	1.33±0.02	1.56±0.05

IL-8, interleukin-8; LECs, lymphatic endothelial cells; OD, optical density.

**Table III:** Effect of IL-8 on LYVE-1, VEGF-D, VEGF-C and VEGFR-3 protein expression.

Group	LYVE-1	VEGF-D	VEGF-C	VEGFR-3
Control	1.00±0.00	1.00±0.00	1.00±0.00	1.00±0.00
0 ng/ml	0.99±0.03	0.99±0.06	1.09±0.03	0.81±0.08^b^
0.2 ng/ml	0.98±0.06	0.84±0.03^c^	0.67±0.04^d^	0.57±0.04^d^
0.5 ng/ml	1.06±0.06	0.83±0.04^d^	0.63±0.09^d^	0.63±0.04^d^
0.8 ng/ml	1.14±0.03^d^	0.82±0.10^d^	0.50±0.07^d^	0.62±0.02^d^
1.0 ng/ml	1.50±0.04^d^	0.59±0.03^d^	0.69±0.03^d^	0.75±0.03

a P<0.05,

b P<0.01 vs. control group,

c P<0.05,

d P<0.01 vs. 0 ng/ml group. IL-8, interleukin-8; LYVE-1, lymphatic vessel endothelial hyaluronic acid receptor-1; VEGF-D, vascular endothelial growth factor-D; VEGF-C, vascular endothelial growth factor-C; VEGFR-3, vascular endothelial growth factor receptor-3.

**Table IV:** Effect of IL-8 on LYVE-1, VEGF-D, VEGF-C and VEGFR-3 mRNA expression.

Group	LYVE-1 mRNA	VEGF-D mRNA	VEGF-C mRNA	VEGFR-3 mRNA
Control	1.01±0.17	1.01±0.19	1.00±0.03	1.00±0.15
0 ng/ml	1.19±0.03	0.62±0.12	0.95±0.07	1.66±0.11^b^
0.2 ng/ml	1.28±0.09	0.58±0.06	0.91±0.09	1.24±0.04^d^
0.5 ng/ml	1.41±0.09^c^	0.41±0.07^c^	0.92±0.08	1.33±0.05^d^
0.8 ng/ml	1.49±0.14^d^	0.24±0.02^d^	0.86±0.05	1.32±0.04^d^
1.0 ng/ml	1.56±0.11^d^	0.33±0.00^d^	1.01±0.04	1.36±0.13^d^

a P<0.05,

b P<0.01 vs. control group,

c P<0.05,

d P<0.01 vs. 0 ng/ml group. IL-8, interleukin-8; LYVE-1, lymphatic vessel endothelial hyaluronic acid receptor-1; VEGF-D, vascular endothelial growth factor-D; VEGF-C, vascular endothelial growth factor-C; VEGFR-3, vascular endothelial growth factor receptor-3.
